# The impact of COVID-19 on the clinical trial

**DOI:** 10.1371/journal.pone.0251410

**Published:** 2021-05-11

**Authors:** Zhimin Chen, Liran Chen, Huafang Chen

**Affiliations:** 1 Ningbo Hospital of Traditional Chinese Medicine, Zhejiang, China; 2 Tang Wealth Investment Management Co. Ltd, Shanghai, China; 3 Office of Drug Clinical Trial Institution, First Affiliated Hospital of Wenzhou Medical University, Zhejiang, China; Faculty of Science, Ain Shams University (ASU), EGYPT

## Abstract

The objective of this study was to explore the impact of the coronavirus disease 2019 epidemic on ongoing and upcoming drug clinical trials. Qualitative semi-structured interviews were conducted with clinical trial staff and clinical trial subjects were surveyed by questionnaire in this study. The results of interviews and questionnaire showed that coronavirus disease 2019 pandemic has led to many changes in the implementation of drug clinical trials, including: a variety of meetings being held online webinars using various platforms, telemedicine and follow-up by video, A large number of deviations from protocol and losses of follow-up, delivery of clinical trial drugs by express, additional workload caused by screening for coronavirus, and anxiety of subjects. These results suggest that the coronavirus disease 2019 outbreak has hindered the progress and damaged the quality of clinical trials. The online meeting, remote follow-up, express delivery of drugs and remote monitoring in the epidemic environment can ensure the progress of clinical trials to a certain extent, but they cannot fully guarantee the quality as before.

## Introduction

The coronavirus disease 2019 (COVID-19) pandemic has had a worldwide impact with serious clinical manifestations including death on the population [1]. The spread of the coronavirus has had a far-reaching impact on the world’s all walks of life [2] including the drug clinical trials. Clinical trial is the most important and necessary part of drug research and development, therefore, different from the diagnosis and treatment process of ordinary patients, the implementation of clinical trials must strictly comply with the trial protocol, ethics and relevant regulations in addition to the common diagnosis and treatment process. During such a large epidemic period, sponsors of clinical trials, investigators in research institutions, subjects (patients involved in clinical trials) and all faced many difficulties, and the working process of drug clinical trials faced multiple negative dilemmas and had to change.

This study focuses on the impact of COVID-19 outbreak on global or domestic multicenter clinical trials that have been carried out and are currently being enrolled and followed up or will be carried out to begin, discusses what changes are certain to occur and whether they will affect the quality, schedule in clinical trials, and safety of subjects; and the paper doesn’t discuss clinical trials of COVID-19 drug study [3].

## Methods

Qualitative semi-structured interviews were adopted to explore for this study [4]. Interviews with staff involved in the clinical trial were conducted at their workplace. This study used purposive sampling strategy. Recruitment started with clinical trial staff involved in different professional sections of First Affiliated Hospital of Wenzhou Medical University, Ningbo First Hospital, and Affiliated Hangzhou First people’s Hospital Zhejiang of University School of Medical, because different hospital and different specialties in general hospitals undertake different types of drug clinical trials, and various specialties can be selected to cover multiple trials. Interviews were conducted with20 investigators, 8 monitors, 12 clinical research coordinators (CRC) who had different practice characteristics and personal characteristics participating in 11 types of drugs for anti-tumor, diabetes, epilepsy, ankylotic spondylitis, acute stroke, hepatitisb, psoriasis, osteoarthritis, nephritis, asthma and analgesia, and 2 ethics committee members. The content of the interview mainly focused on the difficulties encountered in the implementation and promotion of clinical trials and the effect of solutions tried in the context of COVID-19 pandemic.

Another approach to the study was a questionnaire survey. 260 subjects who were participated in different drug clinical trials, including a total of 180 outpatient subjects and 80 subjects of inpatient or hospitalized after COVID-19, were given questionnaires to investigate the impact on their participation in clinical trials during the COVID -19 outbreak. A total of 249 (outpatient 169 and inpatient 80) valid questionnaires were collected from 260 questionnaires with a recovery rate of 95.8%. The 149 subjects who successfully completed the questionnaire included 78 males and 71 females, with different ages (19–75 years), 49 people living alone, 200 people living with their families or friends, living in the distance of 1–300 km from the hospital (99 patients less than 20km and 50 patients more than 200km). Their diseases included tumor, acute infection, stroke, hematological diseases, traumatic surgery and emergency peritonitis surgery, acute gallstone surgery, heart disease, diabetic foot disease, osteoarthritis, skin diseases, kidney disease and kidney dialysis patients, peptic ulcer bleeding, respiratory diseases including asthma and chronic bronchitis, etc. Approximately 60% of the subjects were emergency or severe patients.

This study was reviewed and approved by Ethics Committee in Clinical Research of The First Affiliated Hospital of Wenzhou Medical University, and the informed consent was waived by the ethics committee. This was a non-intervention study. The questionnaires were fully anonymous and no personal identifying information was collected in interviews and questionnaires.

## Results

By sorting out the interview content (as shown in the Table 1) and the recovered questionnaire (as shown in the Table 2), it was found that the implementation of drug clinical trials caused by the epidemic showed the following changes:

**Table 1:** Summary of clinical trial staffs’ interviews.

	Investigators n = 20	monitors n = 8	CRC n = 12
Approbating the convenience of online investigator protocol seminar	20	8	12
Approbating the convenience of online initial training meetings	7	5	5
Approbating the knowledge gained from online meetings	3	4	3
Focusing on the online meeting the whole time	4	5	6
The proportion of the increased workload for screening COVID -19	11–19%	NA	16–31%
The proportion of remote follow-up	9–15%	11–19%	10–16%
Remote follow-up is better than or equal to follow-up at hospital	9	2	7
Praised the quality of follow-up at other hospitals	9	5	8
The proportion of deviation from the trial protocol caused by COVID-19	51–56%	62–66%	55–66%
Worried about changing of the drug delivery process	514	13	13
OVID-19 pandemic have damaged the quality and progress of the trials	20	8	12

**Table 2:** Summary of subjects’ questionnaire survey.

		Subjects(outpatient) n = 169	Subjects(inpatient) n = 80
age	Median ages	48.3(19.0–64.0)	56.1 (26.0–75.0)
Hospital stay days	Median	NA	5.9 (3.0–12)
Living situation	Living alone	19(11.2%)	30(37.5%)
	Living with partner or children	150 (88.8%)	50 (62.5%)
Distance from home to hospital	≤20km	70 (41.4%)	29 (36.3%)
	20-200km	68(40.2%)	32 (40%)
	≥200km	31(18.4%)	19 (23.7%)
caused by COVID-19	Inconvenience to hospital	150 (88.8%)	68 (85%)
	Deviation from the trial protocol	102 (60.4%)	7 (8.8%)
	The idea of giving up clinical trials	17 (10.1%)	2 (2.5%)
	Emergence of anxiety	92 (54.4%)	32 (40%)
During COVID-19	Be isolated	10 (5.9%)	2 (2.5%)
	Follow up at other hospitals	11 (6.5%)	0 (0%)
	Remote follow-up	49(29.0%)	0 (0%)
	Trial drugs sent by express delivery	48 (28.4%)	2 (2.5%)

### Meetings being held online

Changes have taken place since the inception of clinical trial implementation, with a variety of meetings being held online. During the COVID -19 epidemic, people gathering should be avoided. Lectures have rapidly been developed to be delivered online as webinars using various platforms such as Zoom [5]. face-to-face meetings have rapidly evolved into webinars. Various meetings of drug clinical trials, including the investigator protocol seminars involving global or national multicentre investigators, review meeting of the ethics committee, and initial meeting of drug clinical trial begin at sites, all turned into webinars. The meeting mode based on network technology completely replaced the traditional meeting mode during the epidemic, instead of using online meeting as a small offset in clinical trials as in the past.

The work content of the ethics committee is not consistent with the other clinical trial staff, and is relatively independent, so the interview results of the 2 ethics committee members were not listed in Table 1. Both ethics committee members believe that the completeness of the clinical trial project in the online meeting review is not as sufficient as the discussion in the on-site meeting, especially in terms of subject protection.

### Additional workload caused by screening for COVID–19

The additional workload caused by screening for COVID–19 emerged among related staff and subjects. The clinical trial staffs and subjects must be ensured their no COVID-19 infection and won’t infect others [6], are allowed to contact others. Due to the impossibility to detect all asymptomatic carriers and given their contagiousness [7], some test in order to eliminate COVID—19 infections, such as nucleic acid detection, serum antibody test, chest CT examination, lead to increase a lot of work in clinical trials process [8]. All staffs need to rule out infection or a history of close contact patient infected by COVID-19 before they can work with the implementation of clinical trials. Those staffs include investigators, research nurses, drug administrators and clinical research coordinators (CRC). The largest number tests in exclusion COVID-19 infection were obtained from subjects and their accompanying family members, while their nucleic acid detection were required at follow-up intervals of more than 5 days. In addition, nucleic acid, serum antibody and chest CT examination should be strictly performed by all monitors and auditors, because they come from other places with a history of travel, although they will not touch the subjects, they contact with clinical trial staff.

### A large number of deviations from clinical trial protocol

A large number of deviations from clinical trial protocol, violates of protocol and losses of follow up appeared. The blockage of transportation in the residential area and the isolation of subjects with COVID-19 infection or close contact with person infected by COVID-19 all contributed to the failure of subjects to follow the protocol in clinical trial follow-up on time, Consequently, the indexes of efficacy and safety evaluation were missed. 102 out of 169 outpatients had protocol deviations or violations due to the COVID-19 outbreak, compared with 7 out of 80 inpatients, and 17 outpatients even considered abandoning the clinical trial because of the inconvenience caused by the COVID-19 outbreak. During the outbreak period, many investigators made the decision that some subjects could be followed up remotely according to the assessment of the disease type and severity, followed up by video or telephone, and signed the updated version of informed consent remote by video. The questionnaire showed that there was no immediate remote follow-up for inpatients, but 49 of 169 outpatients had remote follow-up. When subjects were followed up remotely, their drugs were delivered by mail. In the questionnaire, 48 subjects out of 169 outpatients received the investigational drug by express delivery. Some subjects were arranged to follow-up at the nearest hospital from residence after the evaluation of disease. The hospitals which complete the follow-up was covered by this clinical trial project or had experience in clinical trials and be evaluated suitable to carry out follow-up mission by the sponsor. At the same time, the sponsor needed to coordinate the clinical trial drug distribution system to allocate the clinical trial drug group (including high, medium, or low dose group, control drug, and placebo) to subjects who have completed follow-up at a different hospital. No matter which hospital completed the follow-up, as well as video or telephone follow-up, must to be recorded in detail and preserved, and it was the same with the delivery use and recovery records of drugs. When the express delivery service was in a stagnant or slow state due to the impact of the COVID-19 pandemic, the deviation from or violation of the clinical trial protocol were bound to increase.

### COVID-19 screening before the subjects’ biological sample is sent out the hospital

Before the each collection of biological sample transported to the central laboratory, the subject’s COVID-19 should be screened to avoid the risk of infection caused by the sample and the disclosure of the information of the sample. At present, most drug clinical trials set up central laboratories to uniformly test the samples of each clinical trial site, so as to unify the test standards and quickly collect data. The blood sample collection of every subject involved multiple blood samples in multiple follow-up cycles, if the follow-up interval was more than 5 days, the subjects should do the COVID-19 screening again and confirm no infection before the biological sample is sent out the hospital.

### Change of subject’s emotions

Anxiety is a common change in the psychological and behavioral patterns of subjects during COVID-19 pandemic [9]. Subjects participating in a clinical trial are required to complete the treatment of disease and the steps in accordance with the clinical trial protocol, who be required to have good compliance with the protocol of the clinical trial, when the inconvenience of transportation or isolation requirement conflict with the compliance with clinical trial protocol, subjects are prone to anxiety and even depression. In the context of the impact of public health emergencies on public mental health [10], from the perspective of social role, investigator plays not only the role of doctor and investigator for professional guidance in medical treatment and follow-up, but also the role of helper in psychological counseling for subjects.

## Discussion

The advantages of online meeting are as follows: 1. it is easy to organize and the progress is faster since the preparation time of the meeting is shortened; 2. the time and expenditure of gathering people who from different regions of the world or China are reduced. In our interview, while all investigators acknowledged the convenience of online meetings, 17of the 20 investigators thought that online investigator meeting did not approbate the knowledge gained from online meetings as the discussion in the on-site meeting, only 3 investigators thinks that the difference was not significant. The 2 interviewed ethics committee members all thought that the online ethics review was not as in-depth and thorough as the on-site discussion. Only 10 (3 investigators, 4 monitors, 3 CRC) Of the 40 interviewees indicated that online investigator protocol seminar and initial training meetings meet the training effects of on-site sessions. 4 of the 20 investigators said they focused on the online meeting the whole time in the interview, another 16 investigators said they would intermittently interject other work during the online meeting. The results of the interviews show that online meetings are not very well received and staffs involved in clinical trials are more likely to accept traditional on-site meetings.

The adaptation of telemedicine methods challenges for investigators. 9 of the 20 investigators said they believed “Remote follow-up is better than or equal to follow-up at hospital “, while the same answer was given by 2 of 8 monitors and 7of 12 CRCs. If you are already familiar with telemedicine methods and have used them in the past, the transition may not be daunting. However, most clinicians have not used telemedicine as a routine part of their daily work. In China, there are not resources that can help clinicians learn about telemedicine, such as “Best Practices in Videoconferencing-Based Telemental Health” [11]. The guidance included in Shore JH ‘s paper [11] was intended to assist in the development and delivery of effective and safe telemental health services founded on expert consensus, research evidence, available resources, and patient needs. In the context of the COVID-19 epidemic, implementing telemedicine services based on this guidance may be one path.

Whether the absence of trial data that due to frequent deviation from protocol or violation of protocol or even loss of follow-up affected the trial quality? Both the staff interview and the subjects’ questionnaire gave consistent responses. 40 staff members believed that the COVID-19 pandemic would damage the quality and slow the progress of the clinical trials. Subjects said the COVID-19 epidemic had made it more difficult than ever to complete all clinical trial process.

Public health emergencies have an impact on the public mental health. In our questionnaire survey, 92 out of 169 inpatient subjects and 32 out of 80 inpatient subjects reported anxiety. As for the emotional changes of subjects caused by anxiety, investigators also have to undertake the task of counseling [12]. The screening of COVID-19, including the sending of biological samples, increases the work difficulty and time of investigators and sometimes even lengthens the trial cycle. Although the application of biological samples transported had rigorously examined and approved by Human genetic resources management office of the Ministry of science and technology [13] before the start of each clinical trial, there are still many loopholes in the management of biological samples, especially those with a particular virus, which should be strictly managed.

In addition, the difficulty of enrolling subjects, the hard times of remote monitoring included the increased workload of CRC in copying and transmitting trial data and the confidentiality of subjects’ information, and the difficulty in implementing the audit, all of which have more or less influenced the quality and process of clinical trials.

Clinical trials need to undertaken carefully, actively and scientifically abiding by the basic principles of“the Helsinki Declaration” [14], despite the urgency generated by the emergence of COVID-19, the investigator must be maintained rigorous evidence and reflect the guidelines for clinical trial basic principles. No matter what the situation, the subject’s safety must be the top priority. Under the epidemic situation, it is an urgent topic for us to explore and discuss to do our best to ensure the safety of the subjects and the quality of clinical trials.

The weakness of this study is that COVID-19 epidemic may not disappear in a short time and human may have several or more years to coexist with it. Subjects, investigators, ethics committee members, monitors and CRCs may gradually adapt to the condition of slight relief and long-lasting persistence of COVID-19, so some of the views in this article will become less obvious over time, such as the anxiety of the subjects. Therefore, follow-up can also be done to explore the management mode of clinical trials in a long time, low incidence rate COVID-19 epidemic situation.

## Conclusion

The COVID-19 pandemic has hindered the progress and damaged the quality of clinical trials. The online meeting, remote follow-up, express delivery of drugs and remote monitoring in the epidemic environment can help the progress of clinical trials to a certain degree, but they can’t guarantee the same quality as before. It is particularly important for the safety and psychological state of the subjects, so investigators may spend more time and energy to ensure the quality and smooth progress of clinical trials in COVID-19 pandemic period.

## Supporting information

S1 File (DOC)

S2 File (DOC)
