# Supplementary material for: The impact of COVID-19 on the clinical trial
Source: PLoS One. 2021 May 11;16(5):e0251410. doi: 10.1371/journal.pone.0251410 (PMC8112689; doi:10.1371/journal.pone.0251410)
Supplement: S1 File — (DOC) [file pone.0251410.s001.doc]

中文问卷调查表（questionnaire in Chinese）：

新型冠状病毒肺炎对临床试验影响的问卷调查表

亲爱的受试者：

您好！

我们正在进行一项关于新型冠状病毒肺炎对临床试验影响的问卷调查，旨在了解新型冠状病毒疫情流行给您参加临床试验带来的不便和影响。本调查采用无记名方式，我们将对您的回答严格保密。对您提供的无私帮助我们表示由衷的感谢。

新型冠状病毒肺炎对临床试验影响研究组 2020年5月

您的基本情况

性别： 年龄: 岁 就诊科室:

您因 疾病参加临床试验。

（请在选择项处划√）

1、您是门诊还是住院就诊：

A、门诊；

B、住院；

2、 如果您是住院病人，您大约需要住院 天？（门诊病人可不填写此项）

3、您家到医院的距离 ?

A、≤ 20 公里

B、20-200 公里

C、≥ 200 公里

4、您是独居还是与家人或朋友住在一起？

A、独居

B、与家人朋友在一起住

5、与平时相比，您觉得疫情期间到达医院方便吗？

A、与平时一样，方便

B、因疫情带来了不便

6、你是否因为疫情影响导致您不能按照医生的要求遵照执行临床试验方案的流程？

A、有过

B、没有

7、你是否因为疫情的不便想过放弃临床试验？

A、有过

B、没有

8、你是否因为疫情被要求隔离过？

A、有过

B、没有

9、你是否因为疫情带来的不便而到就近的医院进行临床试验相关就诊？

A、有过

B、没有

10、你是否因为疫情带来的不便而接受过医生的电话或视频临床试验相关就诊？

A、有过

B、没有

11、你的试验药物是否因为疫情的不便采取过快递寄送？

A、有过

B、没有

12、你是否因为疫情的出现心理问题？

A、有过，

B、没有

13、如果您因为疫情出现过心理问题，请问是哪一类心理问题：

A、焦虑

B、抑郁

C、其他

14、您对疫情期间参加临床试验的建议 ：

。

Questionnaire in English：

The Questionnaire of the impact of COVID-19 on clinical trial

Dear Subject,

We are conducting a questionnaire survey on the impact of COVID-19 on clinical trial, aiming to understand the inconvenience and impact of the COVID -19 epidemic on your participation in clinical trial. This survey is conducted anonymously and your responses will be treated in strict confidence. We express our heartfelt thanks to you for your unselfish help.

The Impact of COVID -19 on Clinical Trial Study Team

May 2020

Your basic information

Gender:

Age:

You visit the  departments of hospital.

You are enrolled in the clinical trial due to diseases.

(Please tick √ in the selection)

1. Are you an outpatient or an inpatient?

A. Outpatient

B. Inpatient

2. If you are an inpatient, how long will you stay in the hospital? days. (Outpatient may not fill this out)

3. How far is the hospital from your home?

A. ≤ 20 km

B. 20-200 km

C. ≥ 200 km

4. Do you live alone, live with family or friends?

A. Live alone

B. Live with family or friends

5. Compared with normal conditions, do you think it is convenient to go to the hospital during the COVID -19 epidemic?

A. Convenient, as usual

B. Inconveniences brought by the epidemic

6. Are you unable to follow the procedures of the clinical trial protocol as required by the doctor due to the COVID -19 outbreak?

A. Yes

B. No

7. Do you want to give up the clinical trial because of the inconvenience of the epidemic?

A. Yes

B. No

8. Have you ever been isolated because of the COVID -19 outbreak?

A. Yes

B. No

9. Did you go to the nearest hospital for clinical trial related visiting and treatment due to the inconvenience caused by the COVID -19 epidemic?

A. Yes

B. No

10. Have you ever received a doctor's telephone or video visiting related to a clinical trial because of the inconvenience caused by the COVID -19 outbreak?

A. Yes

B. No

11. Have you ever sent your clinical trial drugs by express delivery due to the inconvenience of the epidemic?

A. Yes

B. No

12. Do you have psychological problems because of the COVID -19 epidemic?

A. Yes, I have.

B. no

13. If you have ever had a psychological problem due to the COVID -19 epidemic, what kind of psychological problem is it?

A. Anxiety

B. Depression

C. Other

D. No

14. Your recommendations for participating in clinical trials during the COVID -19 epidemic:
