# Supplementary material for: The impact of COVID-19 on the clinical trial
Source: PLoS One. 2021 May 11;16(5):e0251410. doi: 10.1371/journal.pone.0251410 (PMC8112689; doi:10.1371/journal.pone.0251410)
Supplement: S2 File — (DOC) [file pone.0251410.s002.doc]

中文半结构访谈提纲(semi-structured interview outline in Chinese)

主题: 新冠病毒肺炎疫情流行对临床试验的影响

1. 对访谈人员进行系统的培训;
2. 访谈人员要熟悉临床试验及医院工作环境及基本工作流程;
3. 访谈过程中，访谈人员需注意访谈对象特点，对访谈问题的顺序、访谈记录的方式和访谈的时间做灵活调整;
4. 访谈中必须涉及的问题：

4.1：确认被访谈者身份，研究者、CRA或CRC？确认是否疫情期间有试验项目开展或准备开展，所开展试验药物的类型。

4.2新冠肺炎疫情流行防止人员聚集是否对临床试验召开的相关会议出现影响？比如研究者方案讨论会,试验项目启动会。您对线上会议的评价？

4.3 新冠肺炎疫情流行是否增加临床试验的工作量？大约的工作量增加比例？

4.4 新冠肺炎疫情流行对临床试验随访影响，电话或视频随访的效果如何？

4.5新冠肺炎疫情流行对试验药物的分发使用是否有影响？

4.6 是否有因为新冠肺炎疫情流行导致的方案违背，出现比例大约是多少？

4.7 您认为新冠肺炎流行对临床试验的进度有没有影响？

4.8您认为新冠肺炎流行对临床试验的质量有没有影响？

4.9 您本人对对新冠肺炎疫情流行期间，如何更好更快地实施临床试验有何建议？

Semi-structured interview outline in English

Topic: The impact of COVID-19 on on clinical trials

1. Conducting systematic training for interviewers;

2. Interviewees should be familiar with clinical trials and the working environment and basic working process of the hospital;

3. During the interview, the interviewer should pay attention to the characteristics of the interviewee and make flexible adjustment to the order of interview questions, the way of interview recording and the time of interview;

4. Questions that must be covered in the interview:

4.1: Confirm the identity of the interviewee, the [investigator](javascript:;)s, CRA or CRC?

Determine whether a trial program was initiated or planned during the outbreak, and what type of drug was being tested.

4.2 Does the gathering of COVID-19 epidemic prevention personnel have any impact on the meetings related to the holding of clinical trials?

For example, the investigator program discussion, the pilot project kick-off meeting. What do you think of online meetings?

4.3 Does the COVID-19 epidemic increase the workload of clinical trials?

Approximately what is the increase in workload?

4.4 Impact of the COVID-19 epidemic on clinical trial follow-up. How effective is the telephone or video follow-up?

4.5 Does the COVID-19 epidemic have any impact on the distribution and use of the investigational drug?

4.6 Is there any protocol violation caused by the Covid-19 epidemic, and what is the approximate proportion?

4.7 Do you think the prevalence of COVID-19 has any impact on the progress of clinical trials?

4.8 Do you think the prevalence of COVID-19 has any impact on the quality of clinical trials?

4.9 Do you have any suggestions for better and faster implementation of clinical trials during the COVID-19 epidemic?
